# Resolution of C1q deposition but not of the clinical nephrotic syndrome after immunomodulating therapy in focal sclerosis

**DOI:** 10.12860/jnp.2015.11

**Published:** 2015-04-01

**Authors:** Tibor Tibor Fülöp, Éva Csongrádi, Anna A. Lerant, Matthew Lewin, Jack R. Lewin

**Affiliations:** ^1^Department of Internal Medicine, University of Mississippi Medical Center, Jackson, MS, USA; ^2^Department of Medicine, Medical and Health Science Centre University of Debrecen, Hungary; ^3^Department of Anesthesiology, University of Mississippi Medical Center, Jackson, Mississippi, USA; ^4^ProPath, Dallas, TX, USA; ^5^Department of Pathology, University of Mississippi Medical Center, Jackson, Mississippi, USA Case Report

**Keywords:** C1q nephropathy, Cyclophosphamide, Focal glomerulosclerosis, Nephrotic syndrome, Sleep apnea

## Abstract

*Background:* The natural evolution of C1q nephropathy (C1qNP) during immunosuppressive treatment is relatively little studied or understood.

*Case Presentation:* A 30 year-old Caucasian female was referred to us for further management of biopsy-proven C1qNP and severe nephrotic syndrome. Serologic work-up remained negative, including complement C3 and C4 levels and repeated testing for antinuclear antibodies. A renal biopsy revealed minimal change nephropathy vs. focal sclerosis on light microscopy and C1qNP on immunopathology. She has failed trials of high-dose oral prednisone, mycophenolate mofetil 1,500 mg twice a day and a subsequent regimen of monthly IV cyclophosphamide 750 mg × 9 cycles. She also received the maximum tolerated angiotensin-converting enzyme inhibitor and spironolactone therapy. Random urine protein-to-creatinine (UPC) ratio predicted proteinuria in the range between 5-35 gm/day, while serum creatinine rose progressively from 1.0 mg/dL to 1.4 mg/dL (to convert to μmol/L, multiply by 88.4). A decision was made to repeat renal biopsy to reassess the underlying histology. The biopsy revealed focal sclerosis but no C1q deposition.

*Conclusions:* Our case illustrates at least two points: first, an established pathologic diagnosis does not obviate the need for repeated renal biopsy later on, should diagnostic uncertainty persist. Second, histological diagnoses may evolve over time, especially in a patient receiving active and powerful immune-modulating treatment. In our case, the clinical nephrosis did not change with immunosuppressive therapy while C1q deposition ceased, making this latter entity likely the immunologically mediated process.

**Implication for health policy/practice/research/medical education:**

C1q nephropathy (C1qNP) is an uncommon histopathologic finding, with a predominant deposition of C1q in glomerular mesangium. Herewith, we have documented a complete resolution of C1q deposition after immunomedulating therapy, without meaningfully changing the clinical picture of nephrotic syndrome. The overall presentation and clinical course of our patient was compatible with steroid-resistant focal glomerular sclerosis.

## 1. Introduction

The natural evolution of C1q nephropathy (C1qNP) during immunosuppressive treatment is relatively little studied or understood. Existing series on C1qNP are relatively limited and do not provide long-term histological follow-up data.

## 2. Case Presentation

### 2.1. Clinical presentation

**Figure 1:**
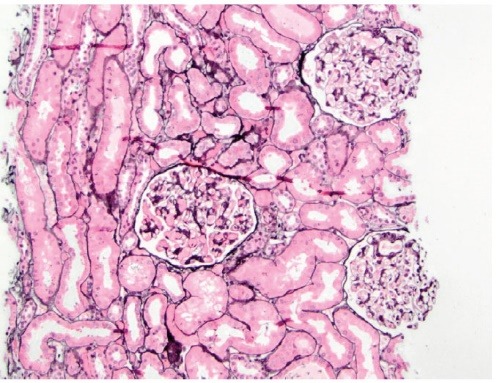
Normal light microscopy, PAMS stain (initial renal biopsy)

**Figure 2:**
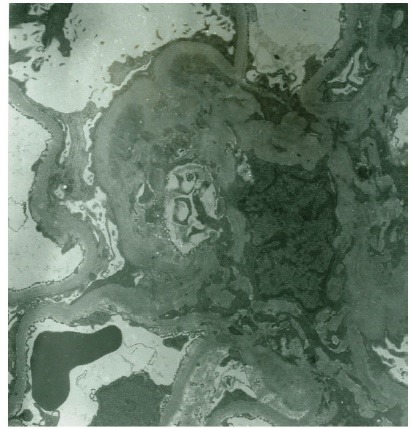
Electron microscopy with mesangial deposits (initial renal biopsy)

**Figure 3:**
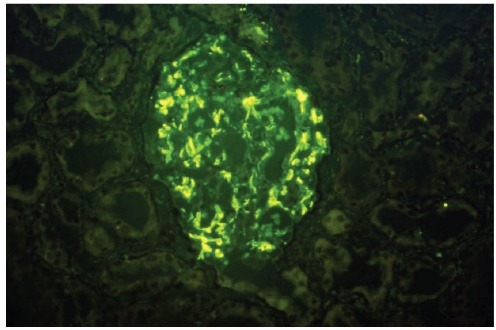
C1q deposition on immunofluorescence (initial renal biopsy).

**Figure 4:**
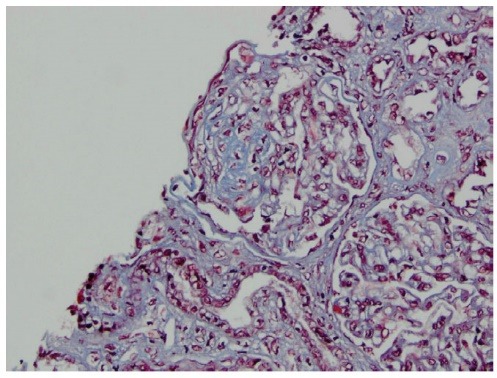
Repeated biopsy with focal and segmental glomerulosclerosis, trichome stain.

**Figure 5:**
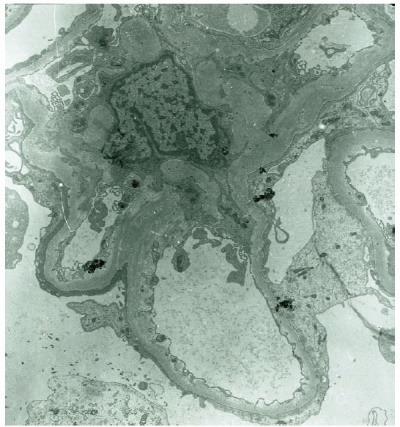
Repeated biopsy with focal sclerosis and no mesangial deposits.

A 30 year-old Caucasian female has been referred to our medical center with a diagnosis of biopsy-proven C1qNP for further management. She had profound nephritic syndrome with serum albumin 1.2-2 g/dL (to convert to g/L, multiple by 10), with co-morbid stage III chronic kidney disease, high blood pressure, untreated obstructive sleep apnea (OSA), hyperlipemia, depression, obesity and electrolyte problems (persisting metabolic alkalosis and hypokalemia). She presented initially to an outside nephrologist with clinical nephrosis 10 months after childbirth with Cesarean delivery. Initial serologic work-up was entirely unremarkable, including serum complement C3 and C4 levels and testing for antinuclear antibodies. One month later (09/2006) she received a renal biopsy revealing minimal change nephropathy versus focal segmental glomerulosclerosis (FSG) on light microscopy and C1qNP on immunopathology. The light microscopy specimen had 10 glomeruli (one of them globally sclerosed) but no fibrosis or crescent formation. Immunofluorescence was also positive for IgG in selected glomerular areas but none was detected for complement C3, C4, albumin or fibrinogen. Electron microscopy described mesangial dense deposits in the glomeruli and an extensive effacement of the visceral epithelial cell foot processes (Figures 1- 3). Thereafter, she received repeated trials of high-dose glucocorticoids (1 mg/kg per os prednisone) via her local nephrologist, but achieved only partial remission (predicted proteinuria range 20-30 g/day) and immediate flares of profound clinical nephrosis, once withdrawal of steroids was attempted. At this point, her primary nephrologist requested a second opinion with our medical center. During her first visit with us (06/2007), she had normal blood pressure of 123/81 mmHg but had severe, 3+ bilateral pitting edema. She has weighed 105 kg and her body mass index calculated at 40 kg/m^2^. Serum albumin was 1.4 gm/dL with random urine protein/creatinine (UPC) concentrations of 4800 and 133 mg/dL (predicted proteinuria 36 gm/day); serum electrolytes were remarkable for serum potassium 3 mM/L and bicarbonate 31 mM/L. A repeated serologic workup with us also remained entirely negative. At that point of time, her medical therapy has been revised with escalation of potassium-sparing diuretics and sleep medicine consultation was initiated for a suspected OSA. Her immuno-modulating therapy was also revised. Her initial regimen was a combination of mycophenolate mofetil 1500 mg twice a day and prednison 40 mg once a day for 14 months, thereafter, we switched to monthly IV cyclophosphamide 750 mg × 9 cycles. Her clinical response to these therapies was only marginal with only mild and transient improvement of nephritic syndrome and subjective well-being. Her care was further complicated by the cessation of compliance with continuous positive airway pressure (CPAP) for OSA and occasional lapses of compliance with medical therapy and follow-up. Altogether, over this period her serum albumin varied from 1.2 to 2.8 mg/dL and predicted proteinuria based on random UPC ranged 5-35 g/day, while creatinine rose progressively from 1.0 mg/dL to 1.4 mg/dL (to convert to μmol/L, multiply by 88.4). However, with a regimen of “triple diuretics” (furosemide, metolazone, spironalactone) we have been able to control clinical nephrosis and peripheral edema to an acceptable degree. Approximately 2 years after her initial visit with us we faced the clinical dilemma of ongoing nephritis-range proteinuria failing maximized and multifaceted medical therapy. While she claimed that she felt better on IV Cyclophosphamide, she did not achieve meaningful remission of nephrosis on any of the attempted immune-modulating therapy, along with maximally tolerated doses of ACE inhibitor and spironolactone. Therefore, a decision was made to proceed with a repeated renal biopsy to reassess the underlying histology. Her medical therapy at this point included furosemide 80 mg once in the morning and once at noon, spironolactone 400 mg daily, amiloride 5 mg daily, metolazone 5 mg daily, lisinopril 40 mg daily, esomeprazole 40 mg daily, paroxetine 40 mg daily, combined estrogen/progesterone hormone preparation for birth control and as needed trazodone at bedtime. A repeated renal biopsy (07/2009) was a limited specimen without overt interstitial fibrosis, but 1 of the 6 recovered glomeruli showed focal sclerosis; however, no C1q deposition was observed (Figures 4-5). Additionally, immunofluorescence of the specimen was negative for C4 and fibrinogen and had only a very sparse staining for glomerular C3 and tubular IgG. Once again, diffuse podocyte foot process effacement was noted on the electron microscopy specimen but no electron dense deposit. Based on this biopsy, further immunosuppressant medical therapy has been abandoned and she was treated with continuing antihypertensive therapy only, including ACE inhibitors and potassium-sparing diuretics.

### 2.2. Clinical follow-up

After the biopsy, she was lost follow-up for 4 months, at which point her serum creatinine has risen to 2.3 mg/dl. While restarting immune-suppressive therapy was entertained, we felt to be of likely little benefit and it was not pursued further. Thereafter, she was lost for further follow-up for another year (12/2010), when serum creatinine rose 2.8 mg/dL, translating into a MDRD formula based estimated glomerular filtration rate of 20 mL/min/1.73 m2. Serum albumin was low at 1.9 gm/dL and she was still severely nephrotic with UPC ratio ~23 but had no clinical uremia. Subsequently, five years after her initial presentation with us (08/2013) she was started on renal replacement therapy with hemodialysis in the context of a pregnancy, which she accepted only three times a week. She delivered a healthy female infant via C-section at 37 weeks of gestation and her post-operative course remained unremarkable.

## 3. Discussion

The key feature of C1qNP is the presence of dominant or co-dominant staining for C1q component on immunofluorescence exam (1). In our case, both biopsy specimen has been processed in our hospital, using identical processing techniques on frozen tissues. Additionally, the resolution of electron-dense mesangial deposit on electron microscopy coincided with the immunofluorescence findings. Therefore, a variation of the processing technique (2) was unlikely to explain the observed phenomenon. Whether C1qNP is a distinct clinical entity from FSG remains uncertain and debated to some degree. It is usually a rare finding, diagnosed in < 1% of kidney biopsies and seen predominantly in children. The prevailing opinion is that C1q NP represent a variant of FSG with a generally favorable prognosis in minimal change disease (3,4) but not in others (5,6). The clinical dilemma of this potential overlap has been noted as far back as 1997 (7). Others have pointed out the potential overlay with past diagnosis of “sero-negative lupus” and the lack of reliable response to immune-modulating therapy (8). The disease may occur in renal transplant recipients but may be missed if immune-fluorescence exams are not performed routinely. However, in renal transplant recipients, it is usually of no clinical consequences (9). Additionally, in renal transplant recipient, the possibility of C1q NP induction by BK virus nephropathy also has been raised (10). It may be coincidental to an unrelated process, such as diabetic nephropathy (11). Ideal treatment is not well defined and spontaneous improvement have been also observed (12). In a small pediatric cohort, disappearance of C1q deposit and emergence of focal sclerosis have already been documented after immunosuppressive therapy (prednisone, cyclosporine-A) (4,13). However, in another report of a single pediatric patient, the clinical improvement correlated with cessation of C1q deposition (14). Anecdotal evidence suggest efficacy of rituxamab (Rituxan®; monoclonal anti- CD 20 monoclonal antibody) in achieving remission in otherwise therapy-unresponsive cases (15).

This case probes a very interesting clinical concept, i.e., whether biopsy-proven renal histological diagnoses remain stable over time. Example given, histomorphology of treated lupus nephritis tends to drift to predominantly membranous form in patients where biopsy was performed for ongoing clinical indication(s) (16). Our patient had clear-cut C1q deposition, along with features of focal sclerosis, on the initial biopsy. Subsequently, she effectively failed trial of immuno-modulating therapy in terms of reducing albuminuria, including high dose prednisone, mycophenolate mofetil and subsequent repeated dose of monthly alkylating agent with I.V cyclophosphamide. While nephrosis did not meaningfully change with immunosuppressive therapy, C1q deposition ceased, making this latter entity likely an immunologically mediated process. We did consider the possibility of obesity mediating focal sclerosis in our patient. However, the diffuse effacement of podocyte foot processes (rather than a focal nature of the lesions), as well as the heavy degree of albuminuria made an obesity-associated process unlikely.

## 4. Conclusions

We have documented clearance of C1q deposition but not the resolution of clinical nephritis syndrome or progressive chronic renal failure in a patient with biopsy-proven C1q nephropathy. Additionally, our case reinforces two additional points. First, an established pathologic diagnosis does not obviate the need for repeated renal biopsy later on, should the diagnostic uncertainty persist. Second, histological diagnoses may evolve over time, especially in a patient receiving active and powerful immuno-modulating medical treatment.

## Authors’ contributions

TF; first author, correspondence, first draft, discussion, finalizing first draft, clinical correlations. EC; clinical correlations, discussion. AAL; histology, preparation of pictures, manuscript review. ML; pathology and immunology. JRL; senior author, pathology correlations, critical review of the manuscript.

## Conflict of interests

The authors have no conflict of interest to declare.

## Funding/Support

None.
